# Editorial: Metabolic and Redox Regulation at the Center of Aging

**DOI:** 10.3389/fragi.2022.858295

**Published:** 2022-03-28

**Authors:** Sruti Shiva, Namakkal-Soorappan Rajasekaran, Bradford G. Hill

**Affiliations:** ^1^ Pittsburgh Heart, Lung, Blood, Vascular Medicine Institute, University of Pittsburgh, Pittsburgh, PA, United States; ^2^ Department of Pathology, University of Alabama-Birmingham, Birmingham, AL, United States; ^3^ Department of Medicine, Division of Environmental Medicine, Christina Lee Brown Institute, Diabetes and Obesity Center, University of Louisville, Louisville, KY, United States

**Keywords:** aging, metabolism, O-GlcNAcylation, electron microscopy, Nrf2, mitophagy, heart

Changes in metabolism and redox state are important contributors to aging and cardiovascular disease. Despite unequivocal evidence that these processes influence aging and cardiovascular biology, it remains unclear how they do so. This research topic on the Metabolic and Redox Regulation at the Center of Aging presents new perspectives and ideas regarding the roles of redox stress in stem cell aging and ketone bodies in the heart, and the influence of O-GlcNAcylation in biology. Also covered are the methods used to visualize and quantify O-GlcNAcylation, mitochondrial morphology, and mitophagy.

We begin this compilation with a comprehensive review of the role of Nrf2 in stem cells and aging (Dodson et al.). In particular, this manuscript highlights central roles of Nrf2 in the control of metabolic and redox pathways in stem cells. Evidence derived from several model systems suggest that Nrf2 maintains lifelong tissue and stem cell homeostasis. Interestingly, Nrf2 levels decline with age, which appears to downregulate redox genes, lead to lower glutathione levels, and increase levels of oxidation products. In stem cells, Nrf2 regulates processes such as quiescence and clonogenic and neurogenic potential. It also interacts with proteins that directly influence aging, such as progerin, and it influences protein quality control, metabolism, epigenetic modifications, and inflammation. The development of safe and effective modulators of Nrf2 activity could help actualize rejuvenation of tissues in older individuals, with the hope of increasing human health and lifespan.

The collection continues with a review by Chu and colleagues on the role of the ketone body, β-hydroxybutyrate, in the heart (Chu et al.). β-hydroxybutyrate (β-OHB) is implicated in energy metabolism in the heart as an alternative fuel source. In particular, circulating β-OHB levels increase in heart failure and its utilization by cardiomyocytes appears to be an adaptive response to stress during heart failure. In addition, β-OHB has several other functions: it is a signaling molecule and can inhibit histone deacetylases; it influences the production of reactive oxygen species; and it has been shown to activate autophagy. β-OHB has also shown the capacity to inhibit the inflammasome, suggesting an anti-inflammatory role. Interestingly, sodium-glucose co-transporter-2 (SGLT2) inhibitors, used to control glucose levels in diabetics, have been shown to increase β-OHB production. Nevertheless, β-OHB appears to be a double-edged sword because long-term elevation of β-OHB seems detrimental. Further knowledge of how ketone bodies influence the heart and whether they could be appropriately timed and dosed in humans are required to determine whether they could be an actionable therapy for heart disease.

If seeing is believing, then high resolution imaging is certainly important for our understanding of biology. In the next contribution to this compilation, Collins et al. provide an in-depth review on the use of transmission electron microscopy (TEM) in cardiovascular biology (Collins et al.). This review exhaustively covers the cardiac ultrastructures that can be visualized with TEM and provides critical information on sample preparation, TEM image analysis, and common technical problems encountered with TEM as well as strategies for improvement. Quite useful in this manuscript are the ways in which TEM has been used to study cardiovascular disease processes. While many studies focus on mitochondrial morphology and cristae density and organization, TEM has also been used to examine numerous processes critical for cardiac health, such as autophagy and mitophagy. Clearly, this manuscript is a “must-read” for anyone considering use of TEM for understanding cardiac biology.

Rounding out our short compilation is an extensive review of protein O-GlcNAcylation in biology and the technical details that influence the ability to detect, quantify, and identify O-GlcNAcylated proteins (Mueller et al.). O-GlcNAcylation is a posttranslational protein modification that is sensitive to nutrient availability and stress, and has been implicated in nearly all disease states. Nevertheless, detecting protein O-GlcNAcylation reproducibly and identifying modified proteins is no walk in the park. This review by Mueller et al. covers standard and advanced techniques for measuring O-GlcNAc and the activities of enzymes that add or remove O-GlcNAc on proteins, i.e., O-GlcNAc transferase and O-GlcNAcase. Also covered are the approaches to detect O-GlcNAcylation using antibody-, click chemistry-, and mass spectrometry-based approaches as well as the numerous studies that have assessed the functional role of O-GlcNAc in mouse models of disease. We classify this manuscript as essential reading for those that wish to start or continue their journey into the fascinating realm of glycobiology.

We hope that this Research Topic provides readers with new insights of the role of metabolism and redox regulation in aging, health and disease and that it stimulates novel ideas and experiments to further advance the field.

